# Acute Chest Syndrome in Children with Sickle Cell Disease: A Narrative Review

**DOI:** 10.3390/children13050670

**Published:** 2026-05-12

**Authors:** Veronica Ramirez, Jules Mercier-Ross

**Affiliations:** 1 Faculty of Medicine, Université de Montréal, Montreal, QC H3T 1J4, Canada; 2 Centre Hospitalier de l’Université de Montréal, Montreal, QC H2X 0C1, Canada

**Keywords:** acute chest syndrome, disease-modifying therapies, sickle cell disease, transfusions

## Abstract

Acute chest syndrome (ACS) is a common pulmonary complication in children with sickle cell disease, defined by a new pulmonary infiltrate on imaging accompanied by fever and/or respiratory symptoms. ACS pathophysiology is multifactorial and incompletely understood, involving vaso-occlusion, pulmonary infarction, inflammation, hypoventilation, and infection—the latter being a frequent trigger in children. While most pediatric cases are mild, ACS can be life-threatening and requires prompt diagnosis and management to prevent progression into respiratory failure. Mild cases are managed with pain control, IV hydration, empiric antibiotics, incentive spirometry, and supplemental oxygen when needed. More severe cases may require simple or exchange transfusion to reduce hemoglobin S levels and limit further vaso-occlusion. ACS is associated with neurologic events and long-term pulmonary complications, making prevention a clinical priority. Disease-modifying therapies include hydroxyurea and chronic transfusion. This review summarizes current evidence on the pathophysiology, risk factors, clinical presentation, diagnosis, acute management, and preventative therapies for ACS in children.

## 1. Introduction

Sickle cell disease (SCD) is one of the most common monogenic disorders worldwide and affects millions of individuals globally [1], with substantial associated morbidity and premature mortality. Despite advances in supportive care, individuals with SCD continue to experience a reduced life expectancy, estimated to be up to two decades shorter than that of the general population [2]. SCD results from an inherited point mutation in the β-globin gene leading to the production of hemoglobin S (HbS), which confers protection against severe malaria in the heterozygous state but causes hemoglobin polymerization under deoxygenated conditions in homozygous individuals [3]. This polymerization results in erythrocyte deformation, increased rigidity, and the characteristic sickle shape. Beginning in early infancy, recurrent erythrocyte sickling promotes vaso-occlusion, chronic hemolysis, and systemic inflammation, ultimately leading to progressive multisystem end-organ damage, most commonly involving the bones, kidneys, brain, retina, endocrine glands, and lungs. Among these complications, pulmonary involvement represents a major contributor to morbidity and mortality in SCD.

First coined by Charache et al. in 1979, acute chest syndrome (ACS) is a complex and heterogeneous clinical entity currently defined as the presence of a new pulmonary infiltrate on chest X-ray in addition to chest pain, tachypnea, wheezing, cough, or a temperature of more than 38.5 °C [4]. ACS will affect nearly half of patients with SCD at least once in their lives [5], and represents the second most frequent cause for hospital admission in children with SCD [6]. ACS is the most common cause of death in SCD [7], with an overall mortality rate of 1.8%, a risk that is disproportionately driven by adults, while children generally experience a milder clinical course [6].

Despite the clinical importance of ACS in pediatric SCD, existing literature remains fragmented, often adult-focused, and somewhat dated, with limited recent pediatric-specific syntheses, highlighting the need for an updated, clinically integrated review. This narrative review summarizes current evidence on the epidemiology, pathophysiology, diagnosis, management, prevention, and long-term complications of acute chest syndrome in children with sickle cell disease.

A literature search was conducted using PubMed for studies published from database inception through April 2026. Search terms included combinations of “acute chest syndrome,” “sickle cell disease,” “children,” “pediatric,” and “paediatric.” Additional searches included terms related to pathophysiology (“infection,” “fat embolism,” “inflammation”), management (“transfusion,” “exchange transfusion,” “antibiotics,” “incentive spirometry”), and prevention (“hydroxyurea,” “chronic transfusion”). Reference lists of relevant reviews, cohort studies, and clinical guidelines were manually screened to identify additional landmark articles. Priority was given to pediatric-specific studies, large cohort analyses, and consensus guidelines, with adult studies included when pediatric data were limited or absent.

## 2. Pathophysiology

**Figure 1:**
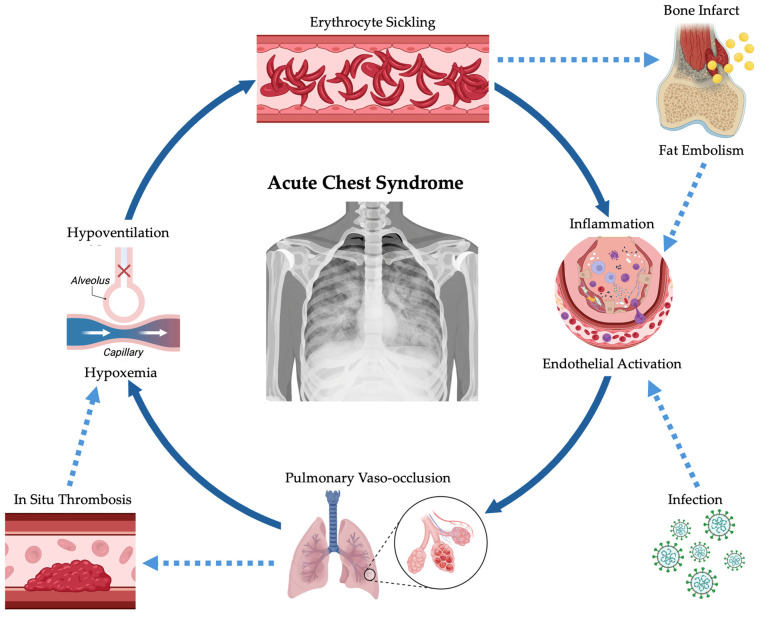
Acute chest syndrome pathophysiology is multifactorial and self-amplifying. Created in BioRender. Ramirez, V. I. (2026) https://BioRender.com/lrbvqf2.

ACS represents a form of acute lung injury specific to sickle cell disease and is multifactorial in origin, as illustrated in Figure 1. Although certain aspects of its pathophysiology remain incompletely elucidated, ACS is widely understood to result from a self-perpetuating cycle of pulmonary vaso-occlusion, regional infarction, inflammation, and alveolar hypoventilation [8]. These processes promote ventilation–perfusion mismatch, progressive hypoxemia, and, in severe cases, acute elevations in pulmonary artery pressures that may culminate in cardiopulmonary instability.

### 2.1. Hypoventilation and Atelectasis

Reduced oxygen tension promotes HbS polymerization and erythrocyte sickling, providing a clear mechanistic link between hypoventilation and the development of ACS. Accordingly, ACS frequently develops in the context of vaso-occlusive pain episodes (VOC), particularly those involving the chest or back, where pain-related splinting leads to decreased chest wall expansion and alveolar hypoventilation [9]. Observational studies have shown that approximately 40% of ACS episodes occur within 2–3 days of hospital admission for VOC, often in the absence of respiratory symptoms at presentation [10,11,12]. In addition, opioid analgesia commonly used for VOC may further depress respiratory drive, exacerbating hypoventilation and promoting regional atelectasis and hypoxemia. These observations provide the physiologic rationale for preventive strategies such as incentive spirometry, which has been consistently shown to significantly reduce the incidence of ACS when used regularly during VOC, beginning with the seminal randomized trial by Bellet et al. in 1995 [13].

### 2.2. Infection

Infection has long been recognized as an important contributor to the development of ACS. In a landmark multicenter study, Vichinsky et al. (2000) [12] identified an infectious pathogen in approximately 30% of ACS episodes, supporting infection as a frequent, though not universal, trigger. Infectious precipitants appear to be particularly relevant in younger children, in whom upper respiratory tract infection symptoms in the days preceding a VOC have been associated with an increased risk of subsequent ACS [14].

Both bacterial and viral pathogens have been implicated, with commonly reported organisms including *Mycoplasma pneumoniae*, *Chlamydia pneumoniae*, respiratory syncytial virus (RSV), *Staphylococcus aureus*, and *Streptococcus pneumoniae* [15]. Seasonal influenza [16] and, more recently, COVID-19 infection [17] have also been associated with ACS.

Importantly, infection alone is insufficient to account for the development of ACS, as respiratory pathogens may be detected in febrile children with sickle cell disease who do not progress to ACS [18]. Moreover, pathogens such as Chlamydia pneumoniae, which typically cause mild respiratory illness in the general pediatric population, have been associated with disproportionately severe pulmonary disease in children with sickle cell disease [19], underscoring the unique host-pathogen interactions that characterize ACS as more than a simple pneumonia. Parvovirus B19 infection, classically associated with aplastic crisis, has also been reported in association with ACS [20], potentially through mechanisms involving bone marrow necrosis and secondary pulmonary fat embolism [21].

### 2.3. Bone Infarction and Fat Embolism

Thoracic bone infarction and subsequent pulmonary fat embolism have been established as a contributor to ACS since the 1970s [22], although this mechanism appears more prevalent in adults than in children with ACS [12]. Fat embolism is thought to promote ACS through a combination of increased red blood cell adhesion, endothelial injury, and stimulation of pro-inflammatory cytokines. In its most severe form, massive pulmonary fat embolism can lead to acute pulmonary hypertension and cor pulmonale, multi-organ failure, and substantial mortality [23].

At the molecular level, fat embolism upregulates vascular cell adhesion molecule-1 (VCAM-1), which promotes erythrocyte adhesion to the respiratory endothelium, leading to increased vaso-occlusion within the pulmonary vasculature and contributing to hypoxia [24]. In addition, embolized fat activates secretory phospholipase A_2_ (sPLA_2_), leading to the release of inflammatory cytokines and free fatty acids that directly injure the pulmonary endothelium and further increase VCAM-1 expression [25]. Circulating sPLA_2_ levels have been shown to correlate with the course and severity of ACS, increasing before symptoms become clinically apparent and normalizing with resolution [26].

Collectively, these processes promote erythrocyte sickling, pulmonary vaso-occlusion, and ventilation-perfusion mismatch, thereby contributing to the development and progression of ACS.

### 2.4. In Situ Thrombosis and Pulmonary Infarction

Pulmonary infarction was identified in 16% of ACS episodes in early multicenter cohort studies [12]. Initially attributed to localized vaso-occlusion leading to hypoxic injury and infarction, subsequent evidence suggests that in situ thrombosis may also contribute. In adult cohorts, imaging has shown that in situ thrombosis is present in up to one in five cases of ACS [27] and is a likely contributor to its development. Although pediatric-specific data remain limited, in situ thrombosis may represent an underrecognized mechanism, particularly in patients without an identifiable infectious or embolic trigger.

### 2.5. Inflammation and Endothelial Activation

Chronic hemolysis in SCD leads to inflammation and ongoing ischemia-reperfusion injuries from microvascular vaso-occlusion, all of which promote endothelial activation [28,29]. Although inflammation alone is unlikely to initiate ACS, it appears to represent a convergent pathway through which diverse triggers—including infection, hypoventilation, fat embolism, and thrombosis—propagate lung injury. Emerging data suggest that complement activation, particularly via the alternative pathway, may contribute to endothelial injury and amplification of inflammation in SCD [30]. Although largely pre-clinical, these findings provide a potential mechanistic link between hemolysis and downstream vascular injury and have generated interest in complement inhibition as a therapeutic strategy. This expanding understanding of inflammatory pathways has also prompted investigation of additional targets, including IL-6 inhibition, with early-phase trials evaluating agents such as tocilizumab.

The complex and multifactorial pathophysiology of ACS is reflected in the variable clinical expression of this disease and helps explain the heterogeneity in its epidemiology and associated risk factors.

## 3. Epidemiology and Risk Factors

While ACS can occur at any age, its incidence peaks between 2 and 5 years of age, with approximately half of children with HbSS experiencing at least one episode of ACS by 6 years old [31]. More recent nationwide analyses confirm that younger age remains an independent risk factor for ACS, as children aged 5–9 experience more than twice the incidence compared to older children admitted with isolated VOC [11]. Furthermore, a first episode of ACS prior to 4 years of age is predictive of recurrent ACS events [32], particularly during the first year following the initial episode [33].

### 3.1. Intrinsic Risk Factors

Beyond age, several intrinsic risk factors have also been associated with increased risk of ACS in SCD patients of all age groups, including HbSS or HbSβ^0^ genotypes, lower fetal Hb concentration, and higher steady-state Hb and leucocyte counts [8,34].

Pulmonary comorbidity represents an important risk modifier in children. Both asymptomatic airway hyperreactivity and asthma are common in children with SCD [11,35]. Children with an established diagnosis of asthma are four times more likely to develop ACS following VOC than those without, and experience longer lengths of stay and higher rates of readmission [5,9]. Emerging evidence suggests that obstructive sleep apnea is also associated with an increased incidence of ACS [11,36].

### 3.2. Acquired Risk Factors

As previously discussed, infection plays a particularly important role in the development of ACS in children. Consistent with this observation, seasonal variation has been reported, with an increased incidence during the viral season in the winter months [6]. Encouragingly, the overall incidence of pediatric ACS has decreased in recent decades, likely due to routine penicillin prophylaxis, expanded pneumococcal immunization, and broader implementation of disease-modifying therapies [37].

ACS can also develop postoperatively, particularly following abdominal surgery, regardless of whether an open or laparoscopic approach is used [38]. The incidence of ACS following abdominal surgery is on the decline [39], possibly due to increased utilization of pre-operative prophylactic transfusion and better post-operative ACS prevention pathways.

Tobacco smoke exposure is a modifiable and clinically actionable risk factor, as it has been shown to increase the incidence of both ACS and VOCs in children with second-hand exposure at home by over 50% [40,41].

The risk factors discussed in this section are summarized in Table 1.

**Table 1:** Risk factors for development of acute chest syndrome in children.

Category	Risk Factor
Patient factors	Age 2–5 years
	Asthma and airway hyperreactivity
	Obstructive sleep apnea
Disease factors	HbSS or HbSβ^0^ genotypes
	Lower fetal hemoglobin
	Higher steady-state Hb and leucocytes
Acquired factors	Viral or bacterial infection Tobacco smoke exposure
	Post-operative state

## 4. Clinical Features, Diagnosis and Predictive Scores

### 4.1. Signs and Symptoms

By definition, to diagnose ACS, the patient must have a new pulmonary infiltrate on imaging in addition to acute respiratory symptoms and/or fever. Importantly, hypoxia is not required, but can be a useful predictor of severity and outcome [42].

Clinical presentation is age-dependent and variable in severity, ranging from a mild self-limited illness to acute respiratory failure requiring intubation. Children up to ten years old, in whom infection is often the trigger, predominantly present with fever, cough, or wheeze [12]. In contrast, fever is less common in older children and adults, most of whom present with dyspnea, chest pain, and VOC in the preceding days [11].

Clinical assessment is unreliable in identifying ACS [43], as approximately 60% of children admitted with ACS can have a normal lung examination at presentation [44]. Depending on severity, intercostal retractions, nasal flaring, and other signs of increased work of breathing can be observed even in older children [21]. When abnormal breath sounds are detected, they are highly suggestive of ACS and may precede any radiographic changes [42]. Clinicians should therefore maintain a high index of suspicion for ACS in patients presenting with respiratory symptoms or signs, particularly in the presence of hypoxia, even when the initial chest radiograph is normal.

Early symptoms may be subtle, and progression from mild respiratory complaints or isolated fever to severe ACS can occur over several hours to days. Approximately 2–5% of pediatric patients with ACS require invasive mechanical ventilation [12,45], although a larger proportion may need monitoring or supportive care in an intensive care unit [46]. Rarely, patients can develop rapidly progressive ACS, leading to respiratory compromise within 24 h and multiorgan failure, possibly in the context of bone marrow necrosis with systemic fat embolism [47]. This ACS phenotype occurs more frequently in adults following VOC but has been described in children [48] and is often preceded by thrombocytopenia.

### 4.2. Investigations

Essential initial investigations should include a chest X-ray (CXR), complete blood count (CBC), hemolysis markers, blood cultures, and screening tests for respiratory viruses, including RSV, influenza, and COVID-19 [42]. Depending on the clinical picture, it may be useful to obtain a hemoglobin electrophoresis and crossmatch if transfusion is being considered. A decrease in Hb relative to baseline in conjunction with increased hemolytic markers is common. In fact, a drop in hemoglobin levels is a significant predictor of impending ACS [49], and the magnitude of Hb decline may correlate with the extent of lung involvement [50] as well as disease severity [48]. Leucocytosis and thrombocytosis are frequently observed [14], whereas thrombocytopenia is also possible and has been associated with increased rates of respiratory failure [12]. However, these laboratory abnormalities are non-specific and must be interpreted in the appropriate clinical context. Notably, detection of respiratory viral pathogens using nucleic acid amplification technique does not reliably predict the development of ACS in febrile children [18].

Measurement of C-reactive protein (CRP) may also be helpful, as recent evidence suggests elevated levels may identify patients admitted for VOC who are at increased risk of developing ACS [51], although further validation is required. Elevated brain natriuretic peptide levels (>30 pg/mL) have been shown to correlate significantly with pulmonary hypertension during severe ACS episodes in adults [10], and may help identify patients at risk for hemodynamic decompensation. Nevertheless, no biomarker has been validated for routine clinical use in diagnosing ACS or predicting disease severity.

D-dimer testing is generally unhelpful if thrombosis is suspected, as it is chronically elevated in the SCD population [42]. Measurement of sPLA_2_ is available only at highly specialized centers and has limited positive predictive value of 24% [52], limiting its current clinical utility.

### 4.3. Imaging Considerations

All febrile SCD patients should undergo chest imaging to evaluate for pulmonary infiltrates or consolidation, even in the absence of respiratory signs and symptoms, given the low sensitivity of clinical examination alone. As previously discussed, CXR abnormalities may lag behind clinical symptoms and repeat imaging may be required to establish a diagnosis. In the face of a completely normal CXR despite clinical suspicion of ACS, high-resolution computerized tomography could be considered, as evidence suggests that this modality has more sensitivity to detect microvascular occlusion not evident on CXR [53]. However, it confers significantly more radiation than CXR and is not routinely recommended as a first line, considering that most patients will require frequent imaging over the course of their life.

Cumulative radiation exposure is a concern as a significant number of patients undergo more than 25 radiographs by adulthood, and 5% of these have over 100 [54]. In this context, point-of-care ultrasound (POCUS) has emerged as a promising radiation-free imaging modality for detecting pulmonary consolidation. Recent studies evaluating POCUS in pediatric ACS have demonstrated negative predictive values exceeding 95%, with some evidence suggesting that ultrasound may detect abnormalities earlier than chest radiography [55,56]. However, its availability and operator dependence may limit widespread implementation.

While not necessary for diagnosis, echocardiography can be useful to detect signs of pulmonary hypertension and right ventricular failure, especially in adolescents who may have baseline cardio-pulmonary dysfunction [8]. Studies have shown that acute pulmonary hypertension is common during severe ACS [57], and that the magnitude of tricuspid regurgitation velocity increase correlates with increased need for invasive ventilation and mortality [10].

### 4.4. Predictive Scores

Given the non-specific clinical features of ACS, and the potential morbidity of a delayed diagnosis, several predictive scores have been proposed, although their performance and utility remain limited. To date, no predictive model has been widely adopted in routine pediatric practice.

Griffin et al. proposed a decision tool to guide chest imaging in febrile children with SCD to reduce unnecessary radiography [58]. The tool incorporates a mix of clinical and laboratory criteria, suggesting the presence of hypoxia, cough, respiratory distress, T > 38 °C, CRP > 50 mg/L or WBC > 15 × 10^9^/L as indications for imaging (see Table 2). The negative predictive value of CXR using the tool was 96%, which has the potential to become clinically relevant, although the tool has not undergone external validation.

More recently, the PRESEV risk score was developed and validated in a multicenter international study involving adult patients from Africa and Europe [59]. This score incorporates readily available clinical variables, including laboratory parameters and pain characteristics (see Table 3), and demonstrated a negative predictive value of 94% for identifying patients at low risk of developing ACS. However, given the distinct pathophysiology and clinical presentation of ACS in children, the applicability of this score to pediatric populations remains uncertain.

## 5. Management of Acute Chest Syndrome

### 5.1. Monitoring and Supportive Care

Patients should be closely monitored for predictors of severe disease, including progressive hypoxemia and respiratory distress, decreasing hemoglobin or platelet count, and multilobar involvement on repeat imaging [42]. Of note, despite previous reports to the contrary, pulse oximetry is considered a reliable measure of arterial oxygen saturation in SCD and is unlikely to underestimate the degree of hypoxemia [60].

Supplemental oxygen should be administered to maintain oxygen saturation >95% or within 3% of the patient’s baseline [4]. Guidelines suggest that vital signs should be monitored at least every 4 h to quickly detect any clinical deterioration [42]. Preliminary evidence suggests that early initiation of non-invasive ventilation (NIV) may prevent deterioration and reduce the need for intubation in patients with severe ACS features, such as severely increased work of breathing, FiO_2_ requirements >50%, or hypercapnia [61]. However, there is insufficient evidence to support this approach, and decisions to initiate NIV should therefore be made on a case-by-case basis in consultation with a critical care team.

Patients with ACS are usually too unwell to maintain oral hydration. Hydration with intravenous fluids at 75–100% of maintenance rate is generally recommended by experts in this context despite paucity of evidence [8,21]. Duration and rate of fluids should be individualized according to the volume and cardiopulmonary status, as these patients are at increased risk of pulmonary edema. Likewise, boluses should generally be avoided unless the patient is hypotensive. There is no guideline recommendation regarding the optimal rate, volume, or type of intravenous fluid.

Adequate pain relief using the World Health Organization analgesic ladder is crucial in the management of ACS, especially in patients admitted with VOC. Undertreated pain can worsen splinting and hypoventilation, thereby contributing to the progression of ACS and potentially leading to further clinical deterioration. Nonsteroidal anti-inflammatory drugs are of particular interest as they do not cause respiratory depression, unlike opioids.

Beyond supportive care, several targeted therapeutic strategies have been investigated to address the underlying mechanisms of ACS.

### 5.2. Antibiotics

Children with ACS should receive empiric antibiotics covering pneumococcus and atypical bacteria, in accordance with current society guidelines. The National Heart, Lung, and Blood Institute (NHLBI) recommends a third-generation cephalosporin combined with a macrolide [4]. Additionally, severely ill patients should receive vancomycin to cover methicillin-resistant *S. aureus* [8].

However, high-quality evidence supporting this approach is limited, as demonstrated by a recent Cochrane review that did not identify randomized controlled trials evaluating antibiotic efficacy in ACS [62]. Current recommendations are therefore primarily informed by commonly implicated pathogens, the diagnostic overlap between ACS and pneumonia, and the potential severity of untreated infection. Although recent evidence suggests that *Mycoplasma pneumoniae* may be less frequently implicated in pediatric ACS than previously thought [18], observational data suggest that children treated with guideline-adherent antibiotics appear less likely to be readmitted than those who received alternative regimens [63]. These somewhat conflicting findings highlight the need for further research to define optimal antibiotic strategies in this population.

In practice, antibiotic selection is often guided by local antimicrobial resistance patterns and patient-specific factors, such as prior colonization with resistant organisms, although direct evidence supporting this approach in ACS is limited.

### 5.3. Transfusion

Beyond simply increasing Hb, blood transfusion increases tissue oxygenation by reducing the proportion of HbS in patients with ACS, and can lead to clinical improvement as early as 12–24 h later [64]. Based on expert consensus, transfusion should be considered early in hypoxic or unstable patients, supported by observational data and a well-established physiologic rationale, although high-quality randomized evidence is lacking.

Based on current guidelines, patients with Hb below 90 g/L and moderate ACS should receive a simple transfusion of 10 mL/kg of red blood cells [65]. One simple “top-up” transfusion is often sufficient to see clinical improvement, but a second transfusion could be considered if patient status is unchanged and Hb level permits it [21].

Exchange transfusion is indicated in patients with features of severe or progressive ACS, such as increasing respiratory distress, oxygen saturation < 90% despite oxygen supplementation, progression of pulmonary infiltrates, or continued Hb decline after simple transfusion [4]. Exchange transfusion should also be considered in patients with high baseline Hb levels precluding simple transfusion [65]. These indications are largely based on expert opinion and observational data. While manual and automated exchange transfusion are considered equivalent, automated exchange will decrease HbS further and more rapidly, which could be relevant in critically ill patients.

Regardless of transfusion modality, all patients should receive extended antigen-matched units to avoid incompatibility with antigens C, E, and Kell at a minimum, and Jk, Fy, and S when possible [65]. Using these precautions, the rate of allo-immunization in ACS has been cited at approximately 2% [12].

Additionally, clinicians should remain vigilant of worsening respiratory status following transfusion, which may be a sign of transfusion-associated circulatory overload or transfusion-related acute lung injury rather than progression of ACS.

### 5.4. Bronchodilators

Bronchodilators have been shown to decrease length of stay in asthmatic patients admitted with ACS, and should be part of standard management in that population [66]. In patients without an established asthma diagnosis, experts suggest a therapeutic trial of bronchodilators if wheezing is present [21], but routine use is not recommended.

### 5.5. Corticosteroids

The role of steroids in ACS management remains controversial due to concerns regarding adverse events. A randomized controlled trial by Bernini et al. [67] demonstrated that using dexamethasone at a dose of 0.3 mg/kg twice daily for 48 h in children admitted with mild to moderate ACS significantly reduced duration of hospitalization, transfusions, oxygen supplementation and total opioid use. This was associated with a trend towards increased readmission for rebound VOC, which was found to be significant in later studies [5,68]. However, the risk for rebound pain appears to be mitigated in patients who receive both corticosteroids and transfusion during their ACS [69]. There have also been reports of serious neurological complications, including hemorrhagic stroke, following corticosteroid use for ACS [70], which makes physicians reluctant to use them in this condition, even in patients with asthma that would likely benefit from their use [71].

Overall, while corticosteroids may improve short-term clinical outcomes, their use requires careful consideration of potential risks. Based on current evidence, experts recommend reserving the use of corticosteroids for children with comorbid asthma [42], and avoiding abrupt discontinuation by using a tapering regimen in order to decrease the risk of rebound pain [25]. The optimal steroid agent, dosing, and duration remain to be determined, highlighting the need for further high-quality controlled studies to better define their role in ACS management.

### 5.6. L-Arginine

Arginine, a semi-essential amino acid, serves as an obligate substrate for the production of the potent vasodilator nitric oxide. Chronic hemolysis in sickle cell disease (SCD) leads to relative arginine depletion, which has been associated with an increased risk of pulmonary hypertension, early mortality, and acute chest syndrome (ACS) [72]. A recent randomized controlled trial demonstrated that intravenous arginine supplementation significantly improved hemodynamic parameters and biomarkers of pulmonary hypertension in children hospitalized with VOC or ACS [57]. However, these physiological improvements did not translate into measurable clinical benefits in that study. Nevertheless, given its favorable safety profile and low cost, arginine supplementation remains an area of ongoing interest as a potential adjunctive therapy for ACS.

### 5.7. Anticoagulation

A recent randomized controlled trial demonstrated that therapeutic anticoagulation reduced the duration of ACS and total opioid use in an adult population [73]. However, these findings cannot be generalized to children given differing pathophysiology, although they may be relevant in older adolescents.

Children with SCD have an increased risk of thromboembolism compared to the general pediatric population [74], particularly adolescents and those with additional risk factors such as central venous catheters or intensive care unit admission [75]. Despite the lack of evidence, the prescription of thromboprophylaxis in hospitalized adolescents with SCD is increasing [76], suggesting that clinicians are increasingly aware of the risk of thrombosis in this subgroup. Evidence supporting prophylactic anticoagulation in this population remains limited, and 2026 guidelines for anticoagulant prophylaxis of pediatric patients do not specifically address SCD. Critical care experts suggest considering thromboprophylaxis in high-risk children with ACS, such as adolescents, those with prior thrombosis, central venous catheterization, COVID-19, or Moya-Moya, following an individualized risk–benefit assessment [77].

## 6. Prevention of Acute Chest Syndrome

### 6.1. Management of Modifiable Risk Factors

Because infection represents a common trigger of ACS in children, strategies aimed at reducing infectious risk are particularly important. These include adherence to guideline-recommended penicillin prophylaxis as well as routine immunizations, including annual influenza vaccination and pneumococcal vaccination [4]. The incidence of pneumococcal-associated ACS has declined substantially following the widespread introduction of pneumococcal conjugate vaccination, paralleling an overall reduction in infectious ACS episodes [78]. Likewise, adequate asthma control and management of obstructive sleep apnea, ideally in collaboration with a pneumologist, may also reduce the incidence of ACS [33].

Incentive spirometry during hospitalization represents one of the few evidence-based interventions proven to reduce the risk of ACS, likely by preventing the hypoventilation and atelectasis that contribute to its development. Incentive spirometry, consisting of 10 sustained inspirations every two waking hours, was first shown to be beneficial in children admitted with chest or back pain [13], with newer evidence suggesting effectiveness in all hospitalized SCD patients, regardless of reason for admission [79]. Despite its demonstrated efficacy, adherence in clinical practice may be variable. More recently, NIV has been proposed as a preventative measure in patients at particularly high risk of ACS, such as those with a history of ACS or sleep apnea, with encouraging preliminary results [80]. However, there is currently no prospective evidence to support its routine use for ACS prevention.

Surgical interventions may precipitate ACS, and evidence suggests that selected patients may benefit from pre-operative transfusion to decrease the risk of perioperative ACS [81]. Current guidelines suggest pre-operative transfusion for procedures requiring general anesthesia lasting over 1 h, using either simple transfusion to raise Hb above 90 g/L or exchange transfusion to achieve HbS below 30% in patients with higher baseline Hb or those undergoing high-risk procedures, such as cardiac or neurosurgery [65]. These recommendations are based on limited randomized data and heterogeneous observational studies, and are largely guided by expert consensus, particularly in higher-risk surgical settings, for which no randomized trials are available.

### 6.2. Disease-Modifying Therapies

Hydroxyurea (HU) is the cornerstone of SCD treatment in patients with severe phenotypes, and current guidelines strongly recommend its use in pediatric patients older than nine months regardless of clinical severity to reduce complications [4]. HU primarily works by inducing fetal Hb, which results in a proportional decrease in HbS, and reduces leucocyte counts and reduces expression of surface adhesion receptors, which may mitigate endothelial inflammation and vaso-occlusion [82]. The BABY HUG randomized controlled trial for HU in infants as young as nine months demonstrated a threefold reduction in ACS compared to placebo over two years, with no detrimental effects on growth or neurodevelopment [83]. Retrospective data suggest improved survival in children treated with HU, primarily due to reduced deaths from ACS and infection [84]. Among available preventive strategies, hydroxyurea has the strongest evidence base for reducing ACS incidence.

In patients who are intolerant of HU or those with recurrent ACS despite maximal tolerated doses, chronic transfusion therapy can be considered to prevent ACS. Retrospective evidence suggests that chronic transfusion in children with a history of severe or recurrent ACS is associated with a tenfold reduction in recurrence rates [85], supporting earlier data from the STOP trial [86]. Despite its efficacy, this treatment strategy is limited by long-term complications with continued use, as chronic transfusion may result in allo-immunization in 15–30% of patients [85], as well as iron overload and, rarely, transfusion-transmitted infections.

L-glutamine, a conditionally essential amino acid involved in redox balance and particularly important in sickle erythrocyte metabolism, has more recently emerged as a potential adjunct to HU therapy in ACS. In a phase III randomized controlled trial, there was a significant reduction in ACS incidence of about 14% in the group receiving oral L-glutamine supplementation after 48 weeks of treatment, with approximately half of participants aged 5 to 18 years old [87]. However, the magnitude of benefit appears modest, and factors such as cost, adherence, and tolerability may limit its widespread use in clinical practice [88].

If ACS recurrence persists despite optimal standard of care, recent guidelines suggest consideration of allogeneic hematopoietic stem cell transplantation [89], as this curative therapy effectively eliminates the recurrence of ACS in successfully engrafted patients [90]. This approach remains generally reserved as a last-line therapy given the considerable acute and chronic transplant-related complications that result in an overall transplant-related mortality of 5% in a pooled analysis [91]. Not all patients may be eligible for a transplant, due to factors such as lack of a suitable donor or older age, as there is a yearly increase in hazard ratio for graft failure or death of 9% [92]. Alternative donor strategies and reduced-intensity conditioning regimens are increasingly being explored, although long-term data remains limited.

Gene therapies have recently emerged as potential curative strategies for SCD and are currently approved for patients ≥12 years with severe sickle cell anemia who are eligible for myeloablative conditioning. However, their role in preventing ACS in pediatric populations remains to be fully established in practice guidelines, and access remains limited to specialized centers [93]. Long-term safety and durability of response remain important areas of ongoing investigation.

In addition to established therapies, several newer agents targeting vaso-occlusion, endothelial adhesion, and inflammation have been developed. Medications such as crizanlizumab and voxelotor have expanded therapeutic options for SCD; however, current evidence does not demonstrate a consistent reduction in the incidence of ACS in clinical trials. This underscores the need for therapies that more effectively target the pulmonary and inflammatory mechanisms underlying ACS.

## 7. Complications and Long-Term Outcomes

While clinicians are widely aware of the significant morbidity and mortality associated with ACS in the acute phase, it is also important to note that its effects on organ function may lead to long-term complications.

The association between ACS and neurological complications is long-established [94], with overt neurologic symptoms reported in up to 8% of pediatric ACS cases [12]. An episode of ACS within the preceding two weeks has been identified as a risk factor for ischemic stroke, as is a history of recurrent ACS [94]. This association may reflect impaired oxygen delivery and systemic inflammatory effects on cerebral blood flow in patients already predisposed to cerebrovascular disease [21]. Following reports of silent cerebral infarcts and reversible posterior leukoencephalopathy syndrome (PRES) in children with severe ACS requiring intubation and exchange transfusion [95], some experts have suggested performing a brain MRI after recovery from severe ACS to screen for occult neurologic injury.

The effects of ACS-related inflammation and vaso-occlusion on lung parenchyma may lead to parenchymal injury, scarring, and fibrosis, which could result in chronic lung dysfunction in both adults and children [96]. Evidence suggests that children who experienced ACS had a greater degree of airflow obstruction than those who did not [96], and that those with ACS recurrence have a higher risk of developing a restrictive lung dysfunction pattern over time [61]. Additionally, it appears that the degree of lung dysfunction in young adults may be proportional to the number of ACS episodes in childhood, with patients who experienced a greater number of ACS episodes having a more severe reduction in lung function testing [97]. That said, multiple studies have reached the exact opposite conclusion [98,99,100], leading some experts to suspect that it is not the quantity but the severity of ACS episodes that explains chronic lung function abnormalities, which remains to be studied [101].

## 8. Conclusions

Pediatric ACS remains a significant source of morbidity affecting a substantial proportion of children with SCD. Symptoms range from mild febrile respiratory illness to severe respiratory failure, reflecting the diverse and complex pathophysiology of this clinical syndrome. Management consists of IV hydration, adequate analgesia, empiric antibiotics, and supplemental oxygen when needed, with a potential role for bronchodilators and corticosteroids in selected patients with comorbid asthma. In more severe cases, simple or exchange transfusion may be required to improve oxygenation and halt disease progression.

Despite advances in supportive care, ACS continues to contribute to both acute complications and long-term pulmonary and neurological morbidity in children with SCD. Early recognition, prompt management, and implementation of preventive strategies—including hydroxyurea therapy, vaccination, and management of pulmonary comorbidities—remain critical to improving outcomes. Continued research into disease-modifying therapies may further reduce the burden of ACS in this population; however, much of the acute management remains supported by limited evidence, and more robust studies are needed to optimize care, particularly with respect to antibiotic selection and predictors of disease severity. In addition, further investigation into emerging mechanisms, such as complement activation and dysregulation, may help identify novel targets for both risk stratification and therapeutic intervention.

## Figures and Tables

**Table 2:** Indications for chest radiography in children from Griffin et al.’s clinical decision tool [58].

Signs or Symptoms	Clinical Features	Laboratory Findings
Respiratory distress	Age < 10 years	CRP > 50 mg/L
Hypoxia	Chest pain	Leucocytes > 15 × (10^9^/L)
Fever > 38 °C	Abdominal or pelvic pain	
Cough	No associated appendicular pain	

**Table 3:** Components of the PRESEV risk score for acute chest syndrome in sickle cell disease.

Variable at Day 1	Points
Reticulocytes (10^9^/L)	
≤216	0
>216	6
Spine and/or pelvis categorial pain scale	
0 or 1	0
2	4
3	6
Leucocytes (10^9^/L)	
≤11	0
>11	3
Hemoglobin (g/L)	
>90	0
≤90	1

A PRESEV score ≤ 5 is predictive of a low risk of developing acute chest syndrome in adults with a 94% negative predictive value.

## Data Availability

No new data were created or analyzed in this study.

## Abbreviations

The following abbreviations are used in this manuscript:

SCD	Sickle cell disease
ACS	Acute chest syndrome
HbS	Hemoglobin S
VOC	Vaso-occlusive pain episode
VCAM-1	Vascular cell adhesion molecule-1
sPLA_2_	Secretory phospholipase A_2_
CXR	Chest X-ray
CBC	Complete blood count
CRP	C-reactive protein
POCUS	Point-of-care ultrasound
NIV	Non-invasive ventilation
HU	Hydroxyurea
